# Dynamics of dimorphic workers of *Constrictotermes cyphergaster* (Blattodea: Termitidae) during nest repair

**DOI:** 10.1093/jisesa/iead118

**Published:** 2024-01-09

**Authors:** Marllon Rinaldo de Lima Andrade, Igor Eloi, Mário Herculano de Oliveira, Maria Avany Bezerra-Gusmão

**Affiliations:** Laboratório de Ecologia de Térmitas, Programa de Pós-Graduação em Ecologia e Conservação, Universidade Estadual da Paraíba, Campina Grande, Paraíba, 58.429-500, Brasil; Laboratório de Biologia Comportamental, Programa de Pós-Graduação em Psicobiologia, Departamento de Fisiologia e Comportamento, Universidade Federal do Rio Grande do Norte, Natal – RN, Brasil; Laboratório de Ecologia de Térmitas, Programa de Pós-Graduação em Ecologia e Conservação, Universidade Estadual da Paraíba, Campina Grande, Paraíba, 58.429-500, Brasil; Laboratório de Ecologia de Térmitas, Programa de Pós-Graduação em Ecologia e Conservação, Universidade Estadual da Paraíba, Campina Grande, Paraíba, 58.429-500, Brasil

**Keywords:** behavior, defense, polyethism, termite

## Abstract

Termite nest repairs are considered a defensive conduct as they reduce the colony’s exposure to the external environment. Repair activities are carried out by worker castes that can be polymorphic, representing a relationship between polymorphism and divisions of functions that can enhance task completion. Repairs are influenced by the extent of damage, nest volume, and the population dynamics of the building species, which regulate the recruitment of individuals for this activity. Our objective was to verify the performances (recruitment for repair) of dimorphic workers of *Constrictotermes cyphergaster* (Silvestri, 1901) during the damage repair activities performed on the external walls of termite nests of different sizes. We found a significant difference in the presence of dimorphic workers that performed repairs, with greater recruitment of the small morphotype, and observed an alternation of morphotypes between initial and final repair activities, with no influence of morphotype on the replacement pattern. Our results also showed that the total number of recruited workers decreased with increasing nest volume. These results help to better understand the social organization of a Nasutitermitinae termite species and the strategies adopted to protect its colonies.

## Graphical Abstract

**Graphical Abstract:**
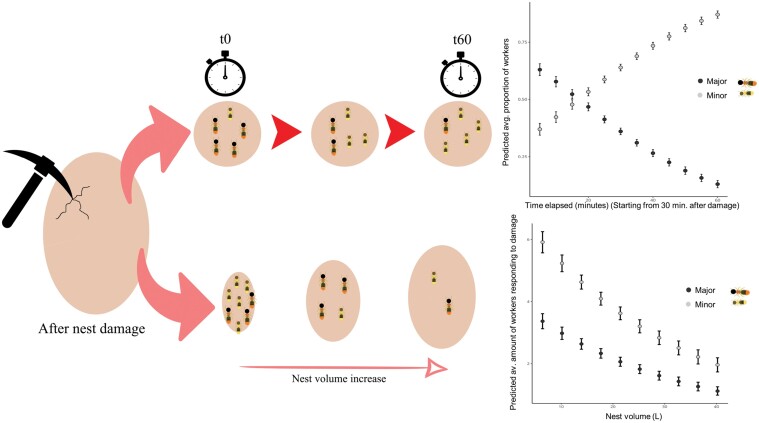

## Introduction

Termites are eusocial insects whose colonies build their own nest systems or can live as tenants in the constructions of other species (Bignell and Eggleton 2000). The nests can be built below or above the ground, and may be composed of a main nest with a system of secondary nests. Nest structures can assume different shapes, sizes, and colors that reflect the life habits of the building species (Ackerman et al. 2007, Sarcinelli et al. 2009). The nest structure, with controlled internal temperature, light levels, and humidity, provides an ideal place for the survival, protection, and maintenance of termite populations (Noirot and Darlington 2000).

Due to these characteristics, nests are characterized as a complementary element in termite defense systems, combined with worker and soldier castes having distinct protective functions, such as patrolling and nest repair that can vary according to nest size (Stuart 1969, Deligne et al. 1981, Anderson et al. 1999, Porter and Hawkins 2001; Šobotník et al. 2008). The influence of nest size on protective activities has been reported in several species of the subfamily Nasutitermitinae and can include the rate of patrolling. An inverse relationship, for example, has been reported between nest volume and the presence of soldiers in a damaged area (DeSouza et al. 2016, Pequeno 2017). This decay in the patrolling rate with increasing nest size may indicate a failure of the colony’s defense system and allow tenants to occupy the largest nests (Oster and Wilson 1978, Muradian et al. 1999, Cristaldo et al. 2012).

Although termite nests are physically resistant, stochastic events or the actions of large predators can damage them. The protection mechanisms of a colony can be enhanced by a division of functions within a colony (polyethism) (Hinze and Leuthold 1999, Soleymaninejadian et al. 2014, Yanagihara et al. 2018). Nest repair is widespread among termites and varies according to the type of construction, the size of the nest, and the magnitude of damage (Bignell et al. 2010).

Workers are the main caste involved in constructing, expanding, and repairing nests, and the workers and soldiers of many termite species show polymorphisms (Miura and Matsumoto 1995, Haifig et al. 2015, Oliveira et al. 2021). Caste polymorphism, combined with polyethism, can be understood as strategies to increase the efficiency of required activities. Studies on the evolution of sociability have reported that phenotypic variability and polymorphism within the same group may have emerged as adaptations for effective group performances (Nonacs and Kapheim 2008), including the productivity of colonies of ants and some subsocial spiders (Pruitt and Riecherter 2011, Modlmeier et al. 2014).

The allocation of different functions among different worker morphotypes has been reported in some termites of the Nasutitermitinae subfamily, such as the tunneling and foraging activities of *Velocitermes heteropterus* (Haifig et al. 2011, 2015), and the foraging activities of *Hospitalitermes medioflavus* (Miura and Matsumoto 1995) and *Constrictotermes cyphergaster* (Oliveira et al. 2021). The relationships between caste polymorphism and nest-building and repair activities, however, are still poorly understood among termites of this subfamily.

We, therefore, used *C. cyphergaster*, which is widely distributed in dry tropical regions such as the Caatinga and Cerrado phytophysiognomies in Brazil (Mathews 1977, Mélo and Bandeira 2004), as a model to examine the dynamics of repair activities and to better understand termite social organization. The nests of this species are built by dimorphic workers using soil particles cemented with saliva. These nest structures will grow linearly in size depending on the size of the population (Vasconcellos et al. 2010).

The examination of nest repair activities carried out by different workers can generate a better understanding of sociability and the integration of colony defenses. Our hypothesis was that the dimorphic workers of *C. cyphergaster* contribute their labors at distinctly different stages during nest repair and that nest volumes influence the presence of individuals in damaged areas. We define “contribute” here as the proportion of workers of a specific size in the same damaged cells at the same time. Thus, we analyzed how the proportions of workers changed over time after nest damage.

## Materials and Methods

### Study Site

**Fig. 1.**
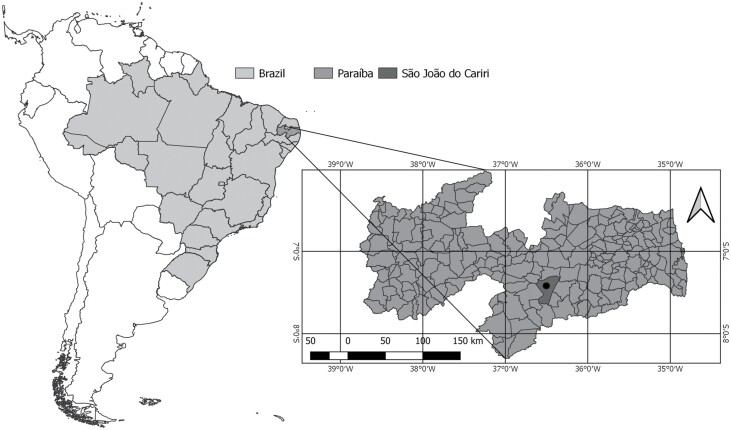
Location of the São João do Cariri Experimental Station (black dot), in the municipality of São João do Cariri, Paraíba, Brazil.

Field observations and termite collections were undertaken at the São João do Cariri Experimental Station (EESJC) (7º20’S, 36º31’W), administered by the Centro de Ciências Agrárias of the Universidade Federal da Paraíba and located in the municipality of São João do Cariri, in Paraíba State, Brazil (Fig. 1). The regional climate is classified as a hot, semi-arid steppe in the Köppen system, with an elevational range of from 400 to 600 m above sea level, and total annual precipitation of 400 mm (Kottek et al. 2006). The landscape is slightly undulating, and the soils are largely classified as Luvisols, Chromic Vertic, Vertisols, and Litholic Neosols. The regional vegetation is dryland shrub Caatinga (Barbosa et al. 2007).

### Nest Repair Observations

Termite nest repair can be divided into 2 stages. During the first stage, the workers block all of the exposed tunnels; the second stage begins after only a few minutes when the workers begin to actively rebuild the damaged outer nest structure (Mathews 1977, Noirot and Darlington 2000). Termites of the Nasutitermitinae subfamily, such as *Nasutitermes coxipoensis* and *Constrictotermes cyphergaster*, can rebuild their galleries within 2 h after being damaged (Bezerra-Gusmão 2008). We therefore define repair activity here as workers effectively repairing an open-cell section of the nest with the deposition of a thin layer of regurgitated material (Noirot and Darlington 2000, Bezerra-Gusmão et al. 2013).

**Fig. 2.**
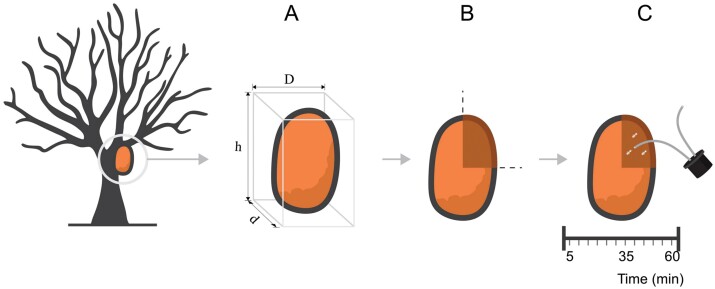
Schematic design of the fieldwork procedure to certify repair activity of the *Constrictotermes cyphergaster*. a) and b) Damage was done to 1/8 of the top surface of the nest. c) Collection of workers involved in the repair activity every 5 min for 1 h, making up a total of 12 sample units per nest.

Observations of repair activities were carried out on 15 arboreal nests of *C. cyphergaster* having different volumes (ranging from 6 to 40 L) during 15 days in January 2020 (observing 3 nests a day) during the dry season. The volumes of the nests were estimated using the formula for hemiellipsoidal objects (*V* = 2/3 πhDd) (Fig. 2a), where *h* = nest height, *D* = 1/2 of its largest diameter, and *d* = 1/2 of its smallest diameter. We inflicted superficial damage on the upper portion of the nest by gently tapping it with a hatchet and then rounding the damaged area to approximately 1/8 of the total outer surface (Fig. 2b).

Prior studies, conducted the previous year with 5 nests of varying sizes (not utilized in the present analyses), revealed that although soldiers respond quickly to damage, workers exhibit a delayed post-damage response, appearing only 25–30 min later. We, therefore, included a 30-min delay between damage and the initiation of nest repair monitoring in this investigation. The nests were subsequently examined for 60 min and the workers observed actively repairing the site were removed every 5 min (Fig. 2c). We carried a total of 180 samples.

**Fig. 3.**
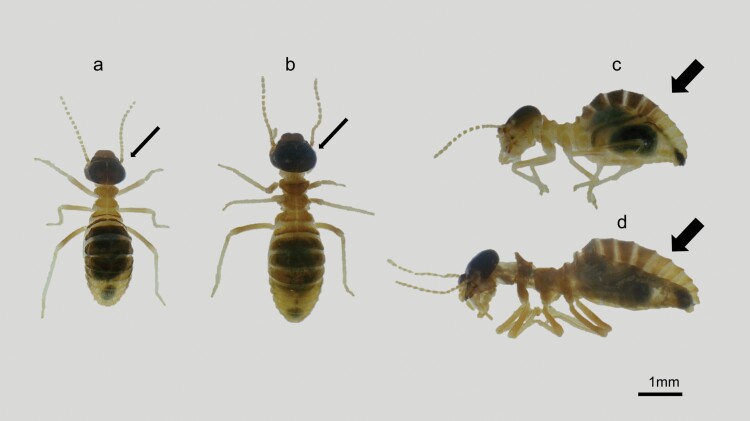
The 2 sub-castes of *Constrictotermes cyphergaster* workers. a) Dorsal view of the minor worker, b) dorsal view of the major worker, c) lateral view of the minor worker, and d) lateral view of the major worker. Thin arrows indicate morphological differences in the head. Note the head capsule is larger and darker in major workers. Thick arrows indicate differences in the curvature of the abdomen. Note that the abdomen is more curved in minor workers.

The workers collected were classified into their appropriate sub-castes (large or small) based on the characteristics listed by Mathews (1977), including the color of the cephalic capsule and body segments (tergites) and the curvature of the abdomen (Fig. 3). Those criteria were used by Oliveira et al. (2021) and adapted for confirming *C. cyphergaster* worker morphotypes. All of the samples obtained were preserved in 96% alcohol and stored as vouchers in the Termite Ecology Laboratory collection.

### Statistical Analysis

The proportions of workers in the damaged area during a sampling unit (y var) were regressed against time and worker morphotype. We built a Generalized Linear Mixed Model with Binomial errors using the glmmTMB package (Brooks et al. 2017). To adjust the model to the influence of morphotype on the proportional response, we included the total number of workers at a given time as an implicit weight. Because our study performed repeated measurements on the same colony, and caste ratios tended to vary depending on nest age (Vasconcellos et al. 2007), we sought to correct our model for these unaccounted variances by including nests as random intercepts.

To determine the most appropriate model, we performed model selection based on Akaike information criterion (AIC), using the MuMIn package (Bartón 2023). The residuals were then inspected using the DHARMa package. Lastly, the model was tested using a type 3 analysis of deviance.

Besides investigating the effects of time and subcaste on the counts of termite individuals, we sought to investigate the influence of nest volume on the damage response. Nest volume can be thought of as a proxy for colony age, and it is therefore fit for indirect testing of age-dependent effects (such as nest resistance and population composition). Because we wanted to treat the number of patrolling workers as a function of nest volume, the rounded collapsed means were calculated to exclude pseudo-replications originating from the timed sampling; a single data point was therefore produced for each of the sampled nests. A generalized linear model was then fitted under Poisson errors, adjusted for underdispersion by specifying a quasi-poisson distribution (assuming that the number of workers is a function of nest volume), with worker morphotypes as co-variables). Model simplification was performed based on the Akaike information criterion (AIC) of competing models, using the DHARMa package. The model was then tested using ANCODEV (analysis of codeviance). All analyses were performed in the R environment (R Core Team. 2022, version 4.2.0).

## Results

A total of 1248 individual termites were collected from the nests (Supplementary Fig. S1); most (63.7%) were small workers (Fig. 5). As an overview, we observed damage response behaviors characterized by an initial flood of soldiers. This explosive reaction lasted approximately 30 min and was closely followed by soldiers being replaced by high numbers of large workers in the damaged area; those large workers gradually decreased in numbers as they were replaced by smaller workers. In the first 5 min (after the initial 30-min waiting period), a mean of 58% (±0.03 standard error) of the individuals found in the damaged area were large workers, with smaller workers accounting for 41% (±0.03). The contingent of large workers began to decrease soon after those first observations. Twenty minutes after initiating monitoring, the large workers represented only 43% (±0.03), and the smaller workers 57% (±0.03). One hour after the start of repair observations, small workers were the absolute majority (78%) (±0.05) in the damaged nest area.

**Fig. 4.**
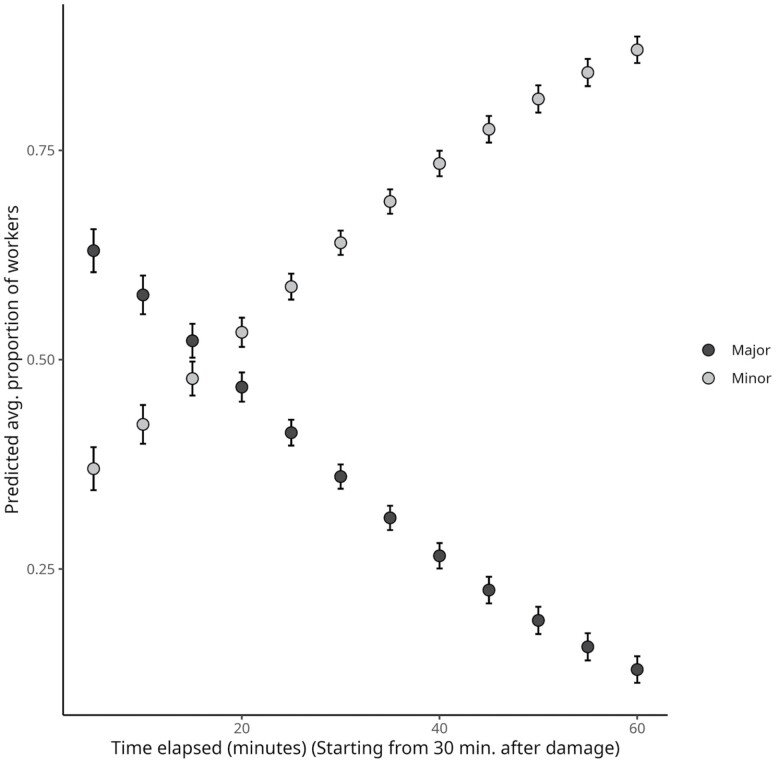
The relationship between the proportion of workers recruited to the damaged surface (*y*) and the duration since the damage (*x*). The values in the plot were estimated via the model, hence each points represents the estimated marginal mean ± the standard error. The substantial interaction term between morphotype and time better describes the pattern of worker turnover at the damage. As time progressed, the originally bigger share of major workers dwindled, with minors accounting for nearly the entire workforce. The model equation is log (proportion of workers) = 0.754531 − 1.509062 × Morphotype:Minor − 0.044278 × Time + 0.088556 × Morphotype:Minor × Time.

The numbers of workers in the damaged area varied proportionally in terms of their morphotypes (GLMM *χ*^2^ = 35.287; *P* < 0.001) and the time elapsed after nest injury (GLMM *χ*^2^ = 126.641; *P* < 0.001), with significant interactions between morphotypes and time (GLMM *χ*^2^ = 253.283; *P* < 0.001) (Fig. 4).

**Fig. 5.**
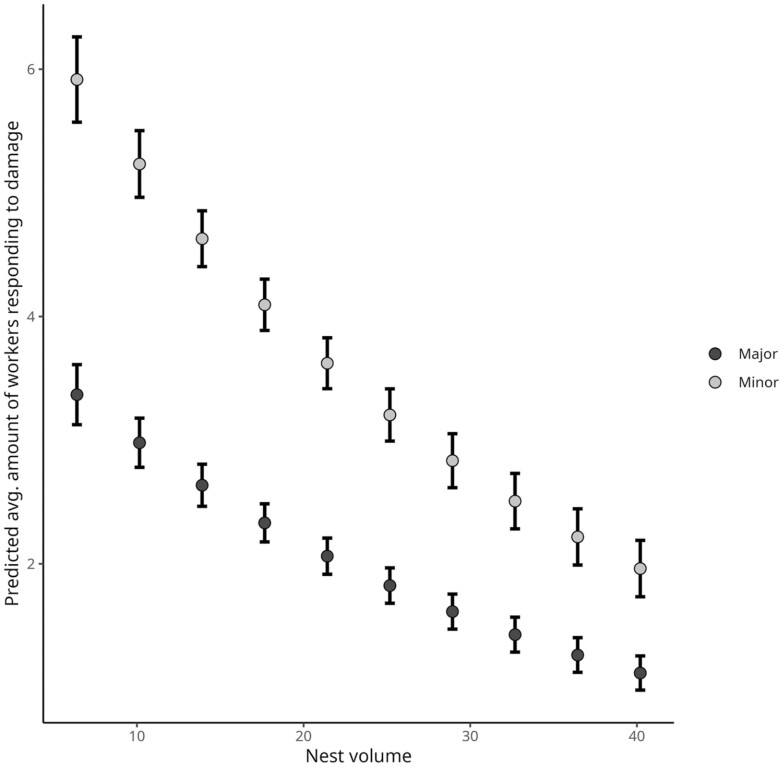
The relation between the number of individuals in the damaged area (*y*), by morphotype, and the nest volume (*x*). The values in the plot were estimated via the model. Each points represents the estimated marginal mean ± the standard error. At the period of an hour, the amount of workers attending the damaged area decreases steadily, regardless of the size of the workers. The model equation is log(average number of individuals) = 1.4233760 − 0.0326675 × nest volume + 0.5634694 × Morphotype:Minor.

In terms of the average number of individuals at the damage site, and taking nest size into account, the pattern of initial recruitment of large workers and their subsequent replacement by smaller workers appears to persist; no negative linear slope was observed, indicating that increasing nest volume reduces the presence of workers in the damaged area regardless of morphotype (Fig. 5). More workers were recruited in response to structural damage in small nests than in larger ones. Increasing nest volume may alleviate the requirement for worker-supported defense responses, and our model predicts a steady decrease in worker defense support as a function of nest volume (GLM *F*_[1,27]_ = 72.119; *P* > 0.001) (Fig. 5). Although small workers outnumber larger workers in damaged regions (GLM *F*_[1,27]_ = 50.4; *P* < 0.001), discrepancies in their proportions are most pronounced in the smaller nests, becoming less pronounced in larger volume nests.

## Discussion

Termite nest repair was described as a defensive behavior pattern by Grassé (1986), with soldiers being the first caste to appear in a damaged area, followed by some workers. Although soldiers are the most specialized caste responsible for colony protection, workers also exhibit defensive behaviors—for example, biting and grasping onto the legs of invaders (Eisner et al. 1976, Prestwich 1984). Those workers also carry out rapid repairs to damaged nest structures, reducing exposure times to the external environment and predator attacks (Noirot and Darlington 2000).

Although this defensive pattern is well-established, the presence of dimorphism among the workers of some Nasutitermitinae termites, such as *C. cyphergaster* (Mathews 1977, Oliveira et al. 2021), can change the dynamics of how those repairs are performed. The recruitment of larger workers for the initial damage inspection behavior and for patching exposed cells corroborates the theory that large workers demonstrate high functional plasticity within colonies of social insects (Gordon 2016, Robinson et al. 2016)—acting like soldiers and thus simulating the defensive behavior of that caste (Noirot and Darlington 2000).

Oliveira et al. (2021) observed that large *C. cyphergaster* workers demonstrate patrolling behavior along the perimeters of foraging trails, suggesting that they have low defense recruitment thresholds. Those same authors reported that large workers were the first to leave the nest during foraging activities and, upon returning to the nest, transmitted vibrational signals to the small workers to consume and store the gathered resources.

As with foraging, signaling and communication between individuals are important to alert the entire colony when facing imminent danger associated with nest disturbances. Cristaldo et al. (2015) observed alarm behavior by *C. cyphergaster,* and reported that soldiers were the first caste to reach a damaged nest region; they demonstrated inspection behavior and used their antennas to evaluate the damaged site. Those soldiers would then emit vibroacoustic signals that initiated the recruitment of other soldiers and some workers. Those authors, however, did not identify the morphotypes of the workers that were initially recruited.

Termites show 2 forms of alarm communication: releasing pheromones and emitting vibroacoustic signals through the substrate (Prestwich 1984, Šobotník et al. 2010). It is known that combinations of these 2 communication forms increase message transmission efficiency and provoke fast colony responses (Hunt and Richard 2013, Cristaldo et al. 2015). The initial exploration patterns of the large workers observed in our study, just after the arrival of the soldiers, can be understood as a consequence of 2 communication forms, with alarm pheromones being produced and secreted from the nasal tip of the soldiers (Deligne et al. 1981) to recruit large workers, which, in turn, emit vibroacoustic signals that recruit smaller workers (Reinhard et al. 2003).

Different from the results reported here, McMahan (1977) and Watson and McMahan (1978) found that large *Drepanotermes rubriceps* and *Nasutitermes exitiosus* workers appeared in greater numbers than smaller workers during nest repair activities. Those large individuals were responsible for the bulk of nest repair work, while the smaller workers only appeared to apply the final touches. Similar to these authors, Zachariah et al. (2017) observed that the repair of *Odontotermes obesus* nests involved large workers depositing large particles in damaged openings (and thus repairing almost all of the breach), while the smaller workers only closed small gaps remaining between the large particles. These results contradict the general notion of a division of labor among termites, with most activities within the colony (such as caring for the offspring and repairing damaged structures) being thought to be carried out by smaller workers; activities outside of the colony (such as foraging and patrolling, on the other hand) are considered to be performed by large workers (Crosland and Traniello 1997).

Dimorphism can be related to both developmental stage and sex in some termite species, with large workers being considered older and female, while smaller workers are generally younger and/or males (Miura 2006). The proximate causes of morphological dimorphism among *C. cyphergaster* workers have not yet been established, however, nor those related to development and gender. Such data would be very useful for understanding whether worker dimorphism in this termite species, combined with the patterns of the division of functions found in our study for repair activity, occurs temporally or sexually.

When analyzing repair activity as related to nest volume, our results showed a very clear trend of the number of workers recruited to the area after damage decreasing with increasing nest volume. Similar results were also reported by DeSouza et al. (2016), who demonstrated reductions in patrolling rates as a function of *C. cyphergaster* nest volume after damage. This reduction in the numbers of both large and small workers during repair activities with increasing nest volume may reflect colony defense system failures. Cunha et al. (2003) and Cristaldo et al. (2012) reported that the presence of cohabitants in *C. cyphergaster* nests was positively related to nest size, and hypothesized that the entry of individual cohabitants into termite nests is facilitated by the favorable conditions of large-volume nests or by the failure of host defense systems to recognize or prevent cohabitation. We did not count the numbers of workers in the nests analyzed in this study but recognize the need for additional research to validate this hypothesis.

Another hypothesis to be raised is that large-volume *C. cyphergaster* nests will have more resistant physical structures due to fecal matter (black mass) accumulations (Cunha and Brandão 2003, Cunha et al. 2003) that can mitigate the likelihood of serious damage to the colony. In addition, as social insect colonies may suffer low-risk disturbances during their growth and expansion processes (Ruiz-Guajardo et al. 2017), those sublethal disturbances may have effects similar to the development of acquired immunity or immunological memory (Sadd and Schmid-Hempel 2006, Pequeno 2017), provoking those (super)organisms to invest more energy in other processes (e.g., the production of alates, migration, or the formation of secondary colonies) (Thorne 1983, Bezerra-Gusmao et al. 2009).

Our results suggest that there are task divisions among the dimorphic workers of *C. cyphergaster* in terms of nest repair activities, with smaller workers constituting the majority of the designated workforce. Only nest volume is negatively related to the presence of these workers during the repair activities. The divergent results reported for other termite species reflect species-specific behaviors adjusted to the contexts in which they are inserted. The life strategies involved in performing certain activities may vary according to the building species, colony stage, and the developmental stages of the individuals performing them. This study shows that the presence of dimorphic workers involved in the repair activities of *C. cyphergaster* nests is dependent on factors such as repair time and colony volume—although we emphasize that there is still no established cause for the pattern found here and that other factors may be related to the dynamics of repair activities by those workers.

The theory of behavioral plasticity in social insects predicts that workers can exhibit task flexibility in response to fluctuating conditions (including their removal during experimental manipulation) (Bonabeau et al. 1996, Theraulaz et al. 1998, Gordon, 2016, Middleton and Latty 2016, Lee et al. 2022). Consequently, the removal of individual workers has the potential to affect the timing of the replacement of larger workers by smaller workers. As such, additional research will be needed to determine exact replacement times using a non-removal methodology.

## Supplementary Material

iead118_suppl_Supplementary_Figures_S1
