# Supplementary figures and images for: Dynamics of dimorphic workers of Constrictotermes cyphergaster (Blattodea: Termitidae) during nest repair
Source: J Insect Sci. 2024 Jan 9;24(1):1. doi: 10.1093/jisesa/iead118 (PMC10776206; doi:10.1093/jisesa/iead118)

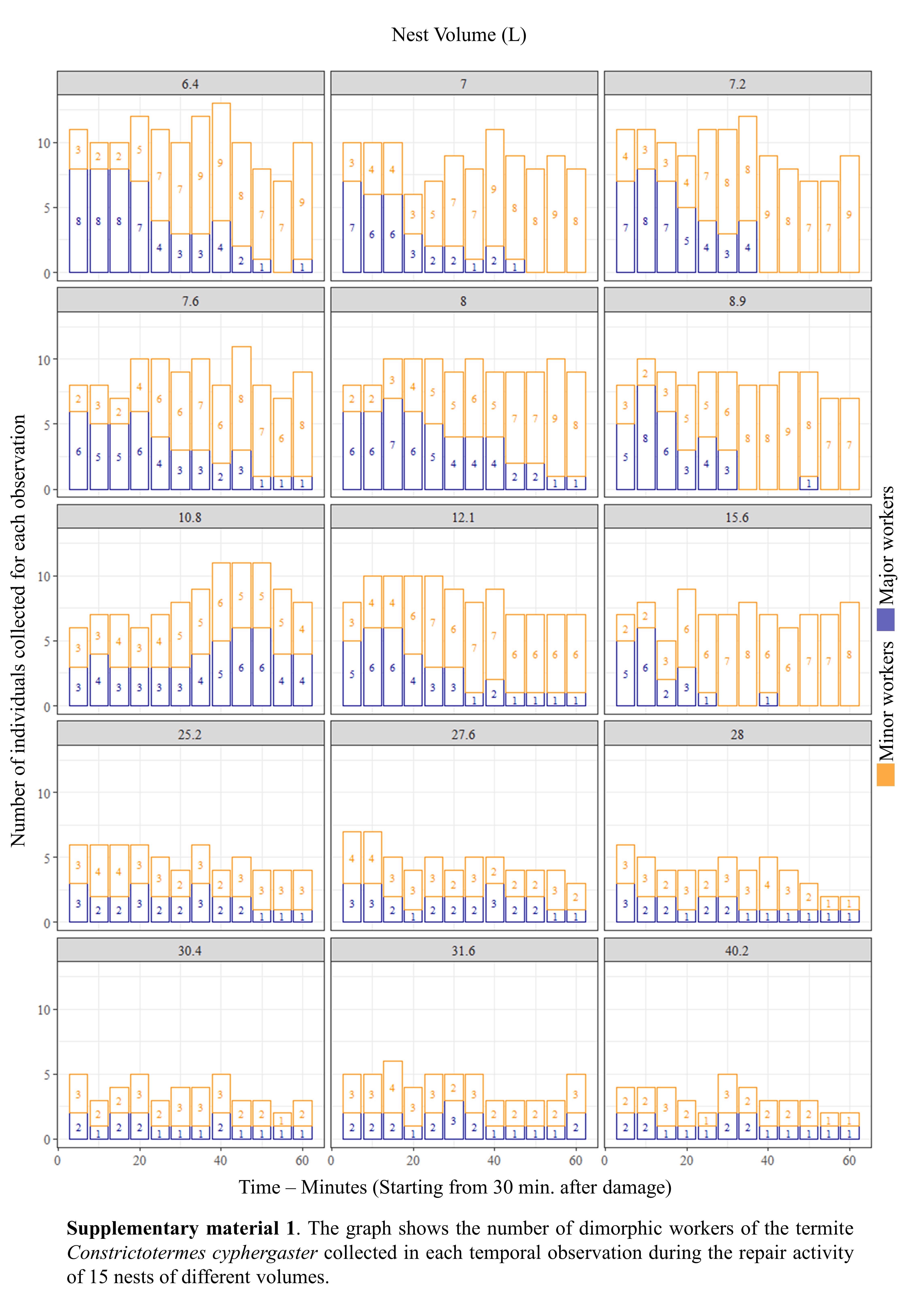

Supplement: iead118_suppl_Supplementary_Figures_S1 [file iead118_suppl_supplementary_figures_s1.jpeg]
